# HOXB4 inhibits the proliferation and tumorigenesis of cervical cancer cells by downregulating the activity of Wnt/β-catenin signaling pathway

**DOI:** 10.1038/s41419-021-03411-6

**Published:** 2021-01-21

**Authors:** Dan Lei, Wen-Ting Yang, Peng-Sheng Zheng

**Affiliations:** 1 Department of Reproductive Medicine, The First Affiliated Hospital of Xi’an Jiaotong University, 710061 Xi’an, Shaanxi People’s Republic of China; 2 Section of Cancer Stem Cell Research, Key Laboratory of Environment and Genes Related to Diseases, Ministry of Education of the People’s Republic of China, 710061 Xi’an, Shaanxi People’s Republic of China

**Keywords:** Cancer, Cell growth

## Abstract

Homeobox B4 (HOXB4), which belongs to the homeobox (HOX) family, possesses transcription factor activity and has a crucial role in stem cell self-renewal and tumorigenesis. However, its biological function and exact mechanism in cervical cancer remain unknown. Here, we found that HOXB4 was markedly downregulated in cervical cancer. We demonstrated that HOXB4 obviously suppressed cervical cancer cell proliferation and tumorigenic potential in nude mice. Additionally, HOXB4-induced cell cycle arrest at the transition from the G0/G1 phase to the S phase. Conversely, loss of HOXB4 promoted cervical cancer cell growth both in vitro and in vivo. Bioinformatics analyses and mechanistic studies revealed that HOXB4 inhibited the activity of the Wnt/β-catenin signaling pathway by direct transcriptional repression of β-catenin. Furthermore, β-catenin re-expression rescued HOXB4-induced cervical cancer cell defects. Taken together, these findings suggested that HOXB4 directly transcriptional repressed β-catenin and subsequently inactivated the Wnt/β-catenin signaling pathway, leading to significant inhibition of cervical cancer cell growth and tumor formation.

## Introduction

Cervical cancer, which is now the fourth most commonly diagnosed type of cancer and the fourth leading cause of cancer-related death among females, causes an estimated 570,000 cases and 311,000 deaths in 2018 worldwide^1^. Notably, it remains to be the second leading cause of cancer-related death in women aged 20–39 years after breast cancer^2^. Human papillomavirus (HPV) infection is clearly a necessary but not a sufficient cause of cervical cancer^1,3^. However, the underlying pathogenesis is still unclear.

The WNT signal transduction cascade orchestrates embryonic development, tissue homeostasis, and regeneration^4^. However, dysregulation of the Wnt/β-catenin signaling pathway is closely associated with various diseases, including cancer^5–8^. Without Wnt signaling, β-catenin can be phosphorylated by a destruction complex (including Axin, APC, PP2A, GSK3, and CK1α), and subsequently degraded by the ubiquitin–proteasome system^8^. Conversely, Wnt binds to its receptors and recruits Axin to the plasma membrane, where it associates with phosphorylated LRP^8^. Subsequently, the destruction complex disrupts, and β-catenin is stabilized^8^. Then β-catenin access to the nucleus, binds to its nuclear partner TCF and upregulates the expression of target genes^8^. Accumulating evidence suggests that the canonical Wnt/β-catenin signaling is involved in the occurrence and progression of cervical cancer^9–16^.

Homeobox (HOX) gene, a subgroup of transcription factors containing a highly conserved homeodomain, has a pivotal role in development^17^. Aberrations of HOX gene expression have been found in cancers including cervical cancer^18–20^. HOXB4, the positive regulator implicated in the self-renewal of hematopoietic stem cells (HSCs)^21^, has been recognized as an oncogene or a tumor suppressor gene, depending on the specific type of cancer. HOXB4 was overexpressed in many cancers, including ovarian cancer^22,23^, cervical cancer^24–27^, lung cancer^28^, renal cancer^29^, mesothelioma^30^, and leukemia^31–34^, which promoted tumor growth and metastasis^23^. Moreover, increased HOXB4 expression resulted in drug resistance^31,35^. In addition, high HOXB4 expression was associated with a significantly shorter overall survival^30^. However, some studies have shown that HOXB4 was downregulated in cancer tissues^36–40^ and reduced in vitro cell proliferation and migration^41^. Other studies focused on the epigenetic alterations, showing hypermethylation of the HOXB4 promoter in cancers^37–40^. Although recent studies have demonstrated that HOXB4 was involved in cervical cancer, its contribution to cervical cancer remains largely unknown.

In this study, HOXB4 was determined to be downregulated in cervical squamous cell carcinoma. Functional studies showed that HOXB4 had a growth-inhibition role in cervical cancer cells. Bioinformatics analyses and mechanistic studies revealed that HOXB4 inhibited cervical tumorigenesis through direct transcriptional repression of β-catenin, which subsequently suppressed the activity of the Wnt/β-catenin signaling pathway.

## Materials and methods

### Tissue specimens

The study was approved by the First Affiliated Hospital of Xi’an Jiaotong University, and all patients were informed of the application of the surgical samples. A total of 97 specimens (including 41 normal cervix, 11 HSIL, and 45 cervical squamous cell cancer) were collected for this study. The histological classification of all primary tissues was confirmed by the International Federation of Gynecology and Obstetrics (FIGO) classification. Fresh specimens obtained during surgery were routinely fixed in formalin and embedded in paraffin or quickly frozen in liquid nitrogen for future RNA isolation and protein extraction.

### Xenograft mouse model

This study was conducted following the guidelines of the Animal Care and Use Committee of the Medical School of Xi’an Jiaotong University. Six- to eight-week-old female BALB/c nude mice were purchased from Shanghai SLAC Laboratory Animal Co., Ltd. Under specific pathogen-free conditions, mice were allowed to eat and drink freely and raised at a constant temperature on a 12-h day-night cycle. Mice were randomly divided into two groups and subcutaneously injected with 1 × 10^6^ cervical cancer cells. At the end of the experiment, mice were euthanized by cervical dislocation. Tumors were harvested and quantified with tumor weight and tumor volume (0.5 × length × width × width).

### Cell culture

All cell lines were purchased from American Type Culture Collection (ATCC) and tested for mycoplasma contamination. Cells were maintained in the recommended media containing 10% fetal bovine serum and 1% penicillin–streptomycin at 37 °C and 5% CO_2_. HeLa, SiHa, C-33A, and 293T cells were cultured in Dulbecco’s modified Eagle medium (Sigma-Aldrich), whereas CaSki cells were cultured in RPMI1640 medium (Sigma-Aldrich). To establish cell lines with stable expression genes or shRNAs, each cell line was transfected with the indicated plasmid using Lipofectamine 2000 reagent (Invitrogen) and selected by G418 (Calbiochem) for 3 weeks. For transient transfection, cells were transfected with plasmids and collected 48 h after being transfected.

### PCR and qRT-PCR

Total RNA was isolated using TRIzol reagent (Invitrogen). Briefly, the sample was treated with 20% chloroform, vortexed, and centrifuged for 15 min. The upper aqueous phase (containing RNA) was collected and an equal volume of isopropanol was added, followed by a centrifugation step for 15 min. The RNA pellet was washed with 75% ethanol, air-dried, and then recovered in RNase-free water. The RNA template was converted into a cDNA using PrimeScript RT Reagent Kit (TaKaRa). The cDNA was then used as a template for PCR amplification using PrimeSTAR HS DNA Polymerase (TaKaRa) or qRT-PCR using SYBR Green Master Mix (Life). Primer sequences were listed in Supplementary Table 4.

### Plasmid construction

All gene expression plasmids were generated by PCR amplification of the cDNA library and cloned into pIRES2-AcGFP-Neo (Clontech) expression plasmids. Short hairpin RNA (shRNA) was used to silence target gene expression and purchased from Shanghai GenePharma Co., Ltd. For the gene promoter dual-luciferase reporter vector, PCR amplification was performed from genomic DNA and then inserted into the pGL3-Basic luciferase vector (Promega). The mutated promoter dual-luciferase reporter vector was established by site-directed mutagenesis. Briefly, the plasmid template was amplified in a mutagenesis reaction with two overlapping primers containing the target mutation site. Then a digestion step by DpnI (NEB) was required to eliminate the original plasmid template. Finally, the recombination reaction and then transformation into *E. coli* competent cells were performed to isolate mutant plasmid. Detailed information regarding primers and oligonucleotide sequences was provided in Supplementary Table 4.

### Immunohistochemistry and immunocytochemistry

Immunohistochemical staining of 5-μm-thick sections was performed using formalin-fixed, paraffin-embedded, and tissue specimens or xenograft tumor samples, according to standard protocols. Briefly, sections were deparaffinized, rehydrated, heated for antigen-retrieval, and pretreated with 3% H_2_O_2_ for 10 min. Sections were preincubated with 10% goat serum to block non-specific binding, incubated with primary antibodies overnight, and then added biotinylated secondary antibody. DAB was used as a chromogen, and hematoxylin was used for counterstaining. The IHC score was calculated by multiplying the staining grade (+0 unstained, +1 weak, +2 moderate, and +3 strong) with the staining ratio of cells (+0 <5%, +1 5–25%, +2 25–50%, +3 50–75% and +4 >75%). A score <3 was negative, while a score ≥3 was positive. For immunocytochemistry, cells grown on coverslips were fixed with 4% paraformaldehyde. After permeabilizing with 0.1% Triton X-100 and blocking with 10% goat serum, antibodies were applied to immunostaining cells. Detailed antibody information was provided in Supplementary Table 5.

### Western blot

Cells were lysed on ice with RIPA lysis buffer pre-added with protease inhibitor Cocktail (Sigma-Aldrich) for 30 min, and then centrifuged at 4 °C for 15 min. Supernatants were measured by protein concentration assay (BCA, ThermoScientific) and then denatured by a 5×SDS loading buffer at 95 °C for 5 min. Nucleoproteins were extracted using Nuclear and Cytoplasmic Protein Extraction Kit (ThermoScientific). Proteins in the cell lysate were separated by SDS–PAGE gel electrophoresis and then transferred to a PVDF membrane. After blocking in 5% non-fat milk for 1 h, the membrane was incubated with primary antibodies under gentle agitation at 4 °C overnight. The membrane was then exposed to HRP-conjugated secondary antibody at room temperature for 1 h and subjected to chemiluminescence using Pierce ECL Western Blotting Substrate (ThermoScientific). Detailed antibody information was provided in Supplementary Table 5.

### Oncomine database analysis

Using the Oncomine (www.oncomine.org) database, the gene expression of HOXB4 in cancer vs. normal tissue was analyzed (*P* < 0.001; fold change > 2).

### RNA-Seq

Sequencing was carried out using the BGISEQ-500 platform at BGI Genomics Co., Ltd. The mRNA was enriched with oligo(dT)-attached magnetic beads. After purification, mRNA was cut into small fragments, and reverse transcription of cDNA was initiated by random hexamers. The A-Tailing Mix and RNA Index Adapters were ligated to the ends of cDNA fragments. The ligation products were then purified and amplified by PCR and validated for quality control. Reads were generated on BGIseq500 platform (BGI) after heating denaturation of PCR products. Using HISAT (http://ccb.jhu.edu/software/hisat), RNA-Seq reads were mapped to the reference genome (human assembly GRCh38.p11). The gene expression level was calculated by RSEM (http://deweylab.biostat.wisc.edu/RSEM). Differently expressed genes can be identified if its expression differed between any two groups with a fold change > 1 and the FDR < 0.01.

### GSEA analysis

TCGA clinical data were derived from The Cancer Genome Atlas (TCGA) (http://cancergenome.nih.gov/), containing 306 cervical squamous cell carcinoma (CESC) patients. Normalized TCGA expression data and RNA-seq gene expression data were analyzed using GSEA software provided by the Broad Institute (http://www.broad.mit.edu/gsea). The enrichment score (ES) was the main result of gene set enrichment analysis, which reflected the degree to which a gene set was over-represented at the top or the bottom of a ranked list of genes. A positive ES indicated that the gene set was enriched at the top of the ranked list and correlated with the first phenotype; a negative ES indicated that the gene set was enriched at the bottom of the ranked list and correlated with the second phenotype.

### Dual-luciferase reporter assay

Dual-luciferase reporter assay was performed using the Dual-Luciferase Reporter (DLR) Assay System (Promega). Briefly, 5 × 10^4^ cells were inoculated into a 24-well culture plate and transiently transfected with 1 mg of the luciferase reporter plasmid and 0.1 mg of the *Renilla* luciferase plasmid (internal control). Cells were collected 48 h after transfection and the luciferase activity was evaluated at 420 nm. Results were shown as the fold change of the experimental group relative to the control group.

### EMSA

Electrophoretic mobility shift assay (EMSA) was carried out using LightShift® Chemiluminescent EMSA Kit (ThermoScientific). Briefly, biotin 5′ end-labeled DNA was heated to 95 °C for 5 min and subsequently with 1 °C min-1 dropped to 4 °C. Typical binding reactions included 20 fmol dsDNA with different concentrations of HOXB4 nuclear protein extracts or 4 pmol unlabeled DNA and incubated at room temperature for 20 min. Reactions were loaded into a pre-run 6% native polyacrylamide gel, electrophoresed in 0.5× TBE buffer, transferred to a nylon membrane, and then cross-linked at a UV light. The bands were detected using chemiluminescence.

### ChIP-qPCR

The chromatin immunoprecipitation (ChIP) assay was performed using the EZ-Magna ChIP Assay kit (Millipore) according to the manufacturer’s instructions. Briefly, cells were cross-linked with 1% formaldehyde for 10 min, quenched with 1× glycine for 5 min, washed with cold PBS, and incubated with a lysis buffer containing protease inhibitors for 15 min on ice. After centrifugation and discarding the supernatant, the cell pellet was collected and then sheared into DNA fragments by sonication. After centrifugation, the supernatant was immunoprecipitated using 5 μg antibodies against HOXB4 (Santa) or IgG (negative control) overnight at 4 °C and pulled down using fully re-suspended protein A/G magnetic beads. Finally, immunoprecipitation was collected, washed, and treated with proteinase K and RNase to purify DNA. The extracted DNA fragments were used as templates for qPCR analysis, and data were normalized with 5% input, respectively. Primers were presented in Supplementary Table 4.

### Cell proliferation and cell cycle assays

Cell proliferation was measured by cell counting, MTT, and colony formation assays. For the cell counting assay, a total of 1 × 10^4^ cells were inoculated in a 6-well culture plate for 7 days and counted every 2 days. For the MTT assay, cells were seeded in a 96-well culture plate at a density of 1000 cells, and 3-(4,5-dimethylthiazolyl)-2,5-diphenyl tetrazolium bromide (Sigma-Aldrich) was added to each well for 7 days. The OD value was measured at 490 nm every 2 days. For the colony formation assay, cells were inoculated in a 6-well culture plate with 1000 cells per well and collected on the 14th day. After washing twice with PBS, cells were fixed with 4% paraformaldehyde for 10 min and then stained with 0.1% crystal violet for 20 min. Visible colonies were counted and photographed. For cell cycle analysis, after synchronizing by serum starvation for 24 h, cells were inoculated in a 6-well culture plate at a density of 6 × 10^5^ per well. Cells were harvested, fixed with 75% cold ethanol, stained with propidium iodide (Sigma-Aldrich) for 30 min, and then run on a FACS Calibur flow cytometer (BD Biosciences) using CellQuest software. Results were analyzed with FlowJo software.

### Statistical analysis

SPSS 20.0 and GraphPad Prism 8.0 software were used for all statistical analyses. Before statistical analysis, data were analyzed for normal distribution and presented as mean ± s.d. Student’s *t*-test was used for comparisons between the two groups unless stated otherwise. *P* < 0.05 was considered significant and shown in the figure. All experiments were performed three or more times with similar results, independently under identical or similar conditions, except stated otherwise.

## Results

### HOXB4 was downregulated in cervical cancer

**Fig. 1:**
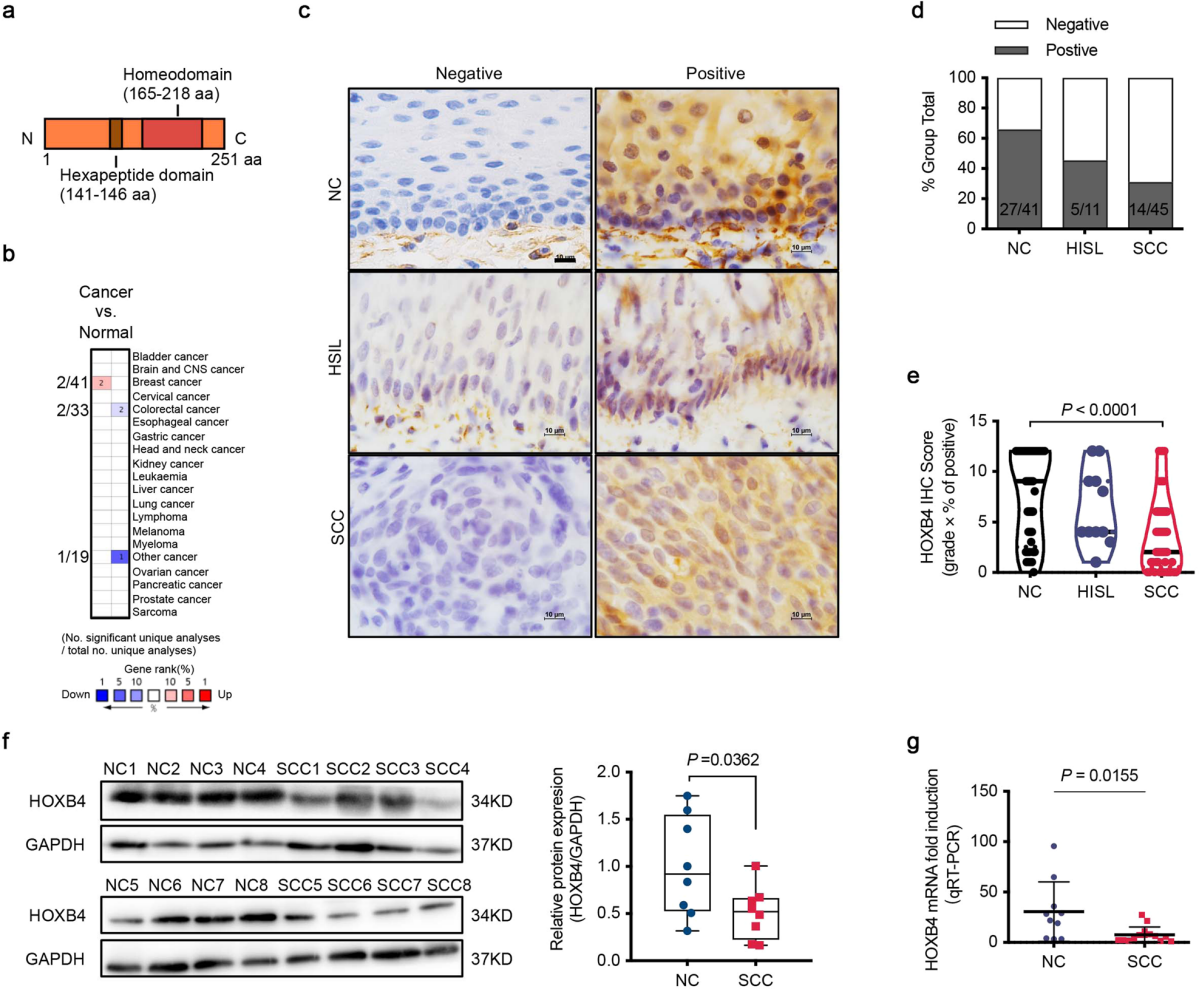
HOXB4 was downregulated in cervical cancer. **a** Illustration of the HOXB4 domains. The relative position of the hexapeptide domain mediating cofactor (PBX) binding and the homeodomain mediating DNA binding were shown. **b** Oncomine analysis of HOXB4 expression in human cancers compared with normal tissues (www.oncomine.org). **c**–**e** Expression of HOXB4 in normal cervix and cervical cancer. Histopathology analysis (IHC staining) of HOXB4 was performed in 41 normal cervix (NC), 11 high-grade squamous intraepithelial lesion (HSIL), and 45 squamous cervical cancer (SCC). Representative images (**c**) with various levels of staining were shown. Scale bar: 10 μm. HOXB4 IHC staining was classified into two categories (negative or positive). Numbers of positive cases were shown (**d**). The relative IHC score (**e**) of HOXB4 was quantified as the product of the grade of staining intensity and the percentage of positively stained cells. One-way ANOVA was used to analyze the data. **f** Expression of HOXB4 in human tissues. Proteins were extracted from eight normal cervix tissues and eight SCC tumors and assessed by WB, with GAPDH normalization (**f**, left). The relative protein expression (**f**, right) of HOXB4 was normalized to that of GAPDH using ImageJ software. **g** HOXB4 mRNA expression levels in ten normal cervix tissues and fourteen SCC tumors and assessed by qRT-PCR. Data were normalized to GAPDH.

HOXB4, a member of the Antp homeobox family, encodes a protein with a homeobox DNA-binding domain, and functions as a sequence-specific transcription factor (Fig. 1a). Oncomine analysis (http://Oncomine.org) showed that HOXB4 was upregulated in breast cancer and downregulated in colorectal cancer, indicating that HOXB4 may have a dual role in cancer pathogenesis (Fig. 1b). To identify the potential role of HOXB4 in cervical cancer, we first performed immunohistochemistry (IHC) in 41 cases of the normal cervix (NC), 11 cases of HSIL, and 45 cases of squamous cell carcinoma (SCC), and tested the expression of HOXB4. The results were classified as negative and positive. Positive HOXB4 staining was observed in the cytoplasm and/or nucleus (Fig. 1c). The percentage of positive HOXB4 staining was 65.9% in NC (27/41), 45.5% in HSIL (5/11), and 31.1% in SCC (14/45) (Fig. 1d and Supplementary Table 1). Moreover, the average HOXB4 IHC score of SCC was much lower than that of NC (Fig. 1e). Additionally, we verified that HOXB4 was downregulated in randomly selected SCC patient tissue samples compared with normal specimens by western blot, which used GAPDH for normalization (Fig. 1f). Moreover, qRT-PCR showed that compared with the normal cervix, the expression of HOXB4 mRNA in randomly selected SCC patient tissues was reduced (Fig. 1g). These data indicated that compared with the normal cervix, the HOXB4 expression was downregulated in cervical cancer.

### HOXB4 suppressed tumorigenesis of cervical cancer cells in vivo

**Fig. 2:**
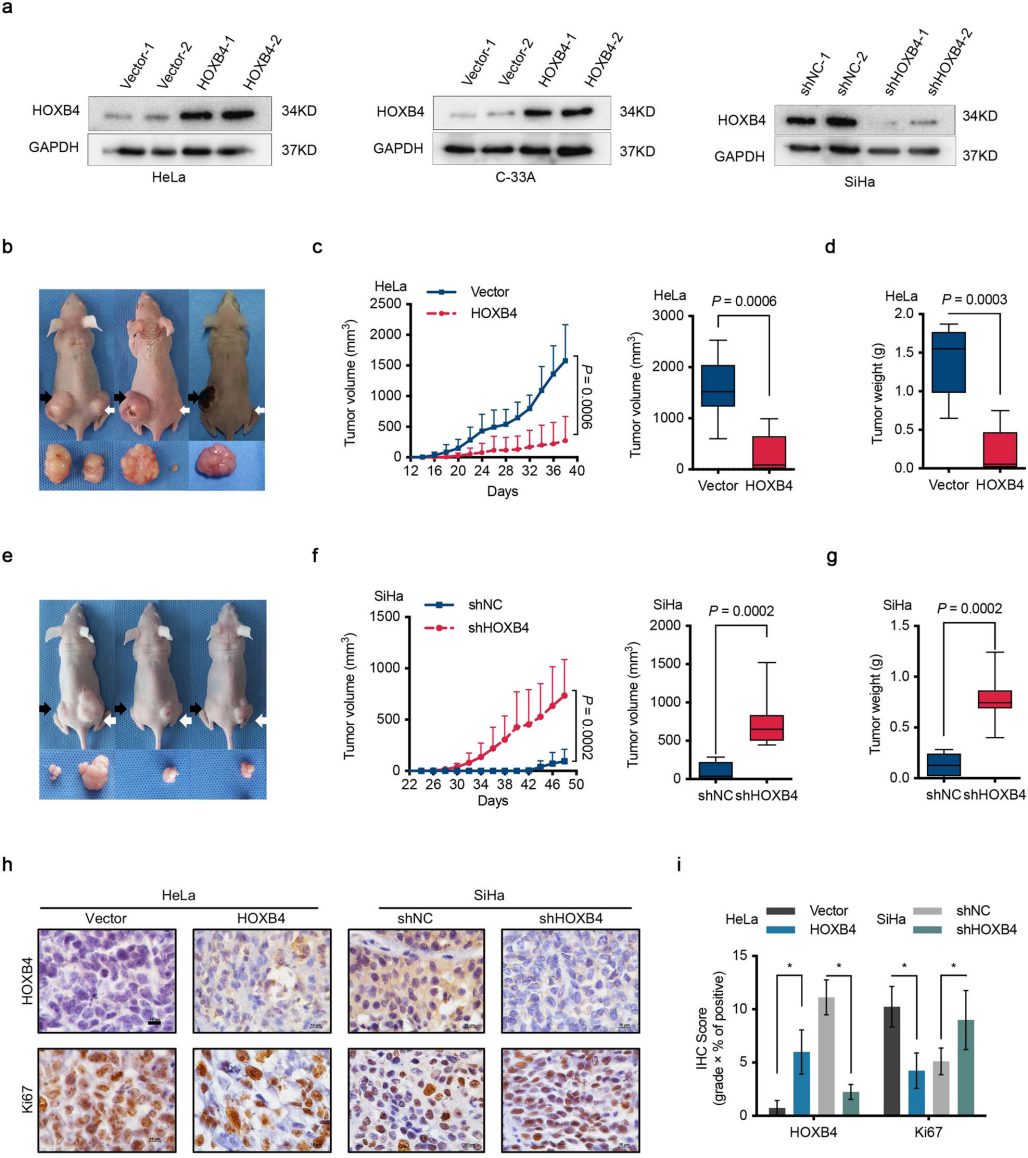
HOXB4 suppressed cervical tumorigenesis in vivo. **a** HOXB4 expression in modified cervical cancer cell lines. HOXB4 protein expression in HeLa (**a**, left) and C-33A (**a**, middle) cells transfected with either empty vector (Vector) or HOXB4 overexpression vector (HOXB4) plasmid. SiHa (**a**, right) cells were transfected with either control (shNC) or shRNAs targeting the HOXB4 (shHOXB4) plasmid and detected using WB. GAPDH was used as a loading control. **b**–**g** Suppression of tumorigenesis by HOXB4. Each mouse (*n* = 8 biologically independent samples) was subcutaneously injected with 1 × 10^6^ HeLa cells into the left (**b** black arrows, Vector) and right (**b** white arrows, HOXB4). SiHa cells were also injected (**e** left: black arrows, shNC; right: white arrows, shHOXB4). At the end of the experiment, mice were killed and tumors were harvested. Representative images of mice and tumors were shown (**b**, **e**). Tumor volumes (mm^3^) (**c**, **f**) and tumor weight (g) (**d**, **g**) were plotted. **h**–**i** Expression of Ki67. Immunohistochemical staining was performed with HOXB4 and Ki67 (**h**) from mouse xenograft tumors and quantification by IHC scores (**i**). Representative images (**h**) were shown. Scale bar: 10 μm.

To elucidate the role of aberrant HOXB4 expression in cervical cancer, we detected HOXB4 mRNA and protein levels in a group of cervical cancer cell lines. Whereas SiHa cells showed abundant expression, HOXB4 was moderately expressed in HeLa, C-33A, and CaSki cell lines (Supplementary Fig. 1a–c). Next, both loss-of-function and gain-of-function of HOXB4 were performed in cervical cancer cell lines. We generated HeLa and C-33A cell lines stably overexpressing HOXB4 or a SiHa cell line with stably knocking down of HOXB4 by two independently targeted short hairpin RNAs (shRNAs) (Fig. 2a and Supplementary Fig. 1d).

To investigate the function of HOXB4 in cervical cancer cells in vivo, nude mice were injected HOXB4-overexpressed HeLa cells and HOXB4-depleted SiHa cells subcutaneously and analyzed for tumor formation. Mice engrafted with HOXB4-overexpressed cancer cells had smaller tumor volumes throughout the experiment than mice engrafted with control cells (Fig. 2b). At 38 days after tumor cell implantation, we observed a 94.4% decrease in the size and a 96.5% decrease in the weight of the tumors formed by HOXB4-overexpressed HeLa cells compared with those of the control tumors (Fig. 2c, d). Notably, HOXB4 overexpression significantly extended the tumor-free survival of animal (Supplementary Fig. 1e). On day 48, compared to the control mice, mice implanted with HOXB4-depleted SiHa cells showed larger tumor volumes, with a 17.1-fold increase in tumor size and a 5.8-fold increase in tumor weight; these mice also had shortened tumor-free survival (Fig. 2e–g and Supplementary Fig. 1f). In addition, the results showed that the IHC score for Ki67 staining was significantly decreased in the tumors stably overexpressing HOXB4, but markedly increased in the tumors with stable knockdown of HOXB4, compared with control tumors (Fig. 2h, i), demonstrating that HOXB4 suppressed tumorigenesis by inhibiting cervical cancer cell proliferation in vivo. Consistent with our observation of in vivo experiments, IHC staining of primary tumors indicated that HOXB4 expression was negatively correlated with Ki67 in cervical cancer patients (Supplementary Fig. 1g, h).

### HOXB4 inhibited the proliferation of cervical cancer cells and arrested the cell cycle in vitro

**Fig. 3:**
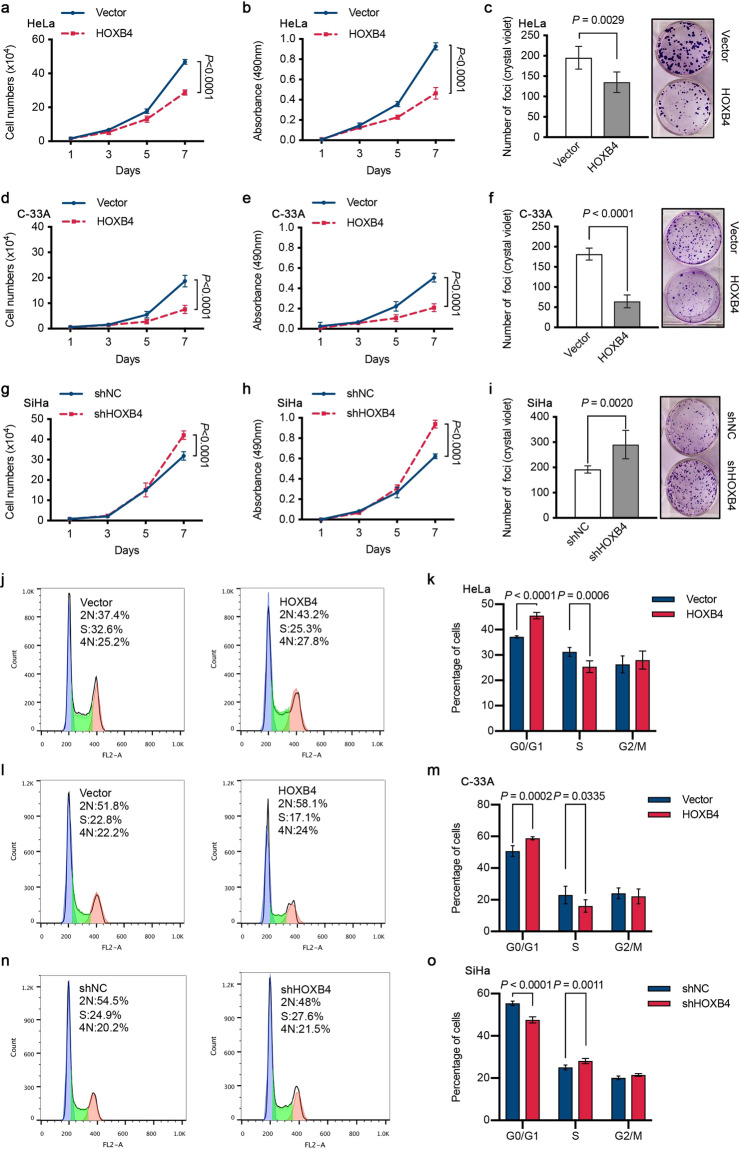
HOXB4 inhibited cell proliferation and arrested the cell cycle in vitro. **a**–**i** Inhibition of cell proliferation by HOXB4. Cell counting (**a**), MTT (**b**), and colony formation (**c**) assays were performed in HOXB4-overexpressing HeLa cells and control cells. Representative images of the colony formation assay with crystal violet staining (**c**, right) were shown, and colonies were quantified by counting (**c**, left). These proliferation experiments (cell counting, MTT, and colony formation assays) were also performed in HOXB4-overexpressing C-33A cells (**d**–**f**) and HOXB4-depleted SiHa cells (**g**–**i**). **j**–**o** Cell cycle distribution in modified transfected cervical cancer cell lines. Cell cycle profiles were obtained by FACS analysis of propidium iodide-stained cells and analyzed by flow cytometry in HOXB4-overexpressing HeLa (**j**, **k**) and C-33A (**l**, **m**) cells, as well as in HOXB4-depleted SiHa (**n**, **o**) cells.

To further verify whether HOXB4 inhibited cervical cancer cell growth in vitro, we performed cell counting, MTT, and colony formation experiments. Compared with the control, the overexpression of HOXB4 in the HeLa cell line significantly reduced cell proliferation and decreased colony formation (Fig. 3a–c). The inhibitory effect of HOXB4 on cell proliferation was further confirmed in the C-33A cell line (Fig. 3d–f). Conversely, HOXB4 knockdown in the SiHa cell line markedly increased cell growth and colony formation relative to control shRNA (Fig. 3g–i). Taken together, these data indicated that HOXB4 inhibited the growth of cervical cancer cells in vitro.

To explore the mechanism of HOXB4 inhibiting the proliferation of cervical cancer cells, flow cytometry was used to analyze the cell cycle of cells stained with propidium iodide. The data showed that, compared with control cells, HeLa cells with continuous upregulation of HOXB4 caused an increase in the percentage of cells in the G0/G1 phase and a decrease in the percentage of cells in the S phase (Fig. 3j, k). Similar results were also observed in C-33A cells (Fig. 3l, m). However, down-regulation of HOXB4 accelerated the G1/S transition, reduced the ratio of G0/G1 phase SiHa cells, and increased the ratio of S phase cells (Fig. 3n, o). Collectively, the cell cycle profiles suggested that HOXB4 inhibited the G1/S transition.

### HOXB4 downregulated the activity of the Wnt/β-catenin signaling pathway

**Fig. 4:**
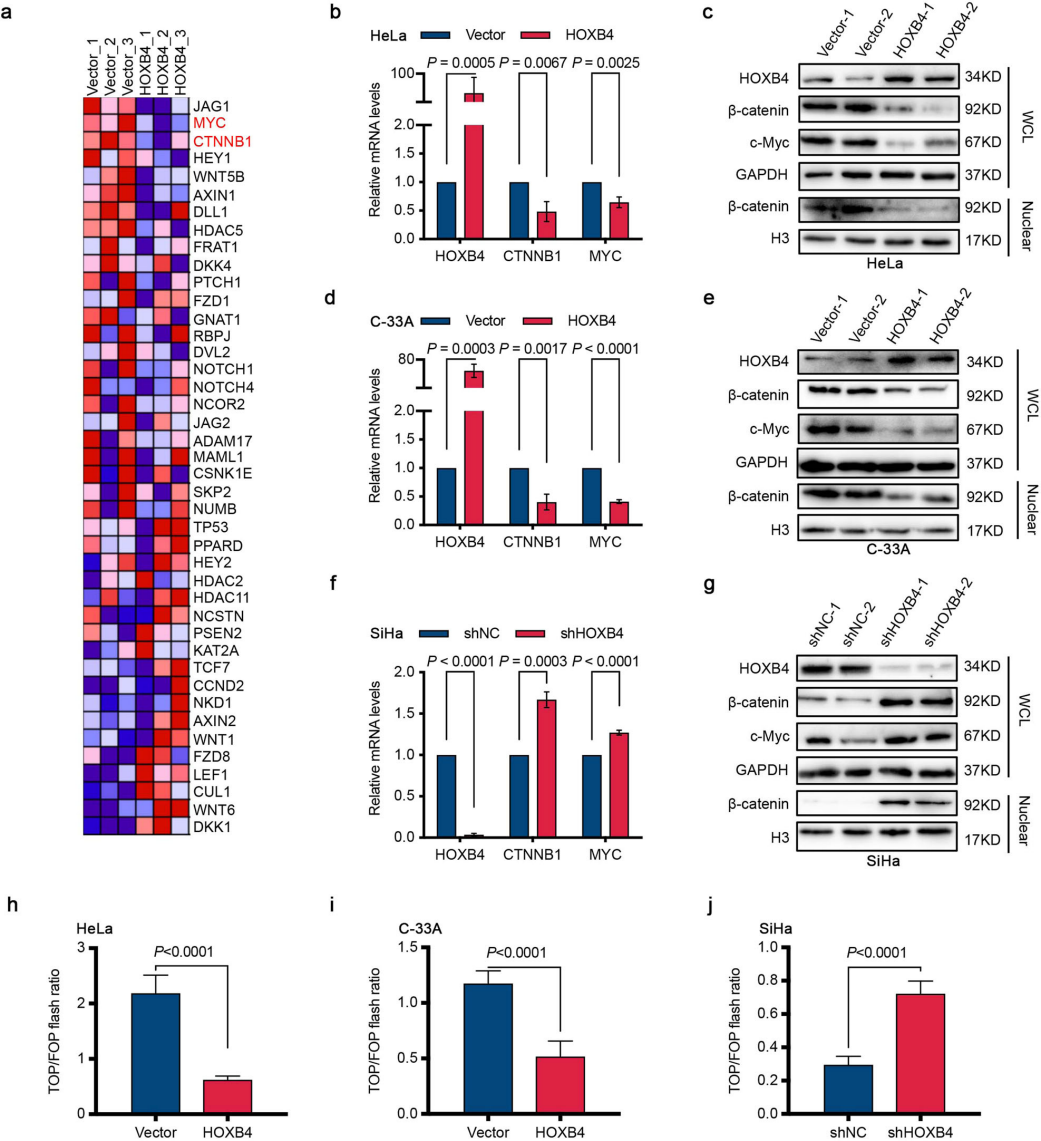
HOXB4 inactivated the Wnt/β-catenin signaling pathway. **a** Heat map of HOXB4 RNA-seq results. All enriched features of the enriched gene sets, which were filtered and analyzed by GSEA analysis of HOXB4 RNA-seq data, were shown between the vector control (Vector) and HOXB4-overexpressing (HOXB4) HeLa cells. Gene expression values were represented as colors, where the range of colors (red, pink, light blue, dark blue) showed the range of expression values (high, moderate, low, lowest). *n* = 3 per group. **b**–**g** Expression of β-catenin and c-Myc. The mRNA levels of HOXB4, β-catenin, and c-Myc were detected by qRT-PCR in HOXB4-overexpressing HeLa (**b**) and C-33A (**d**) cells, as well as in HOXB4-depleted SiHa (**f**) cells. Data were normalized to GAPDH. The protein expression of HOXB4, total β-catenin, nuclear β-catenin, and c-Myc was assessed in HOXB4-modified HeLa (**c**), C-33A (**e**), and SiHa (**g**) cells by western blot and normalized to GAPDH and histone H3 (H3) respectively. **h**–**j** Inactivation of the Wnt/β-catenin reporter luciferase by HOXB4. HeLa (**h**), C-33A (**i**), or SiHa (**j**) cells transfected with the indicated plasmids were analyzed by β-catenin reporter luciferase assays (TOPFlash (TOP) and FOPFlash (FOP)).

To explore the molecular mechanism of HOXB4 in cervical cancer cells, RNA sequencing (RNA-seq) analysis was performed using three HeLa cell lines with stable overexpression of HOXB4 and three control cell lines. A total of 17,894 genes were detected. Gene set enrichment analysis (GSEA) results of six samples revealed that 28/50 gene sets were upregulated by phenotype Group_Vector and 22/50 gene sets were upregulated by phenotype Group_HOXB4 (Supplementary Table 2). Of these gene sets, changes in the Wnt/β-catenin signaling pathway were identified after modification of HOXB4. CTNNB1 and MYC are two major molecules in the Wnt/β-catenin signaling pathway^4^, which were significantly decreased due to the overexpression of HOXB4, in the HeLa cell line, as shown in our RNA-seq results (Fig. 4a and Supplementary Table 3).

To further verify the result indicated by the bioinformatics analysis of RNA-seq, quantitative reverse transcription-polymerase chain reaction (qRT-PCR) and western blot experiments confirmed that HOXB4 overexpression reduced the mRNA and protein levels of β-catenin and the target gene c-Myc, in HeLa and C-33A cells (Fig. 4b–e and Supplementary Fig. 2a, b). Consistent with the down-regulation of total β-catenin levels, the nucleoprotein level also decreased in the HeLa and C-33A cell lines where HOXB4 was overexpressed (Fig. 4c, e and Supplementary Fig. 2a, b). In contrast, knocking down of HOXB4 in the SiHa cell line resulted in β-catenin accumulation and increased c-Myc expression, both at the mRNA and protein levels (Fig. 4f, g and Supplementary Fig. 2c). Likewise, the β-catenin level in the nuclear fraction of SiHa cells knocked down by HOXB4 was increased compared to control cells (Fig. 4g and Supplementary Fig. 2c). In addition, in 293T cells, overexpression of HOXB4 suppressed the expression levels of β-catenin and c-Myc (Supplementary Fig. 2d, e).

The TOP/FOP flash luciferase reporter assay was used to detect the activity of the Wnt/β-catenin signaling pathway, showing an approximately 71.6% reduction and 56.0% reduction in the activity of the β-catenin reporter (TOP flash) in HeLa and C-33A cells stably overexpressing HOXB4, respectively, compared with the control cells (Fig. 4h, i). In contrast, HOXB4 knockdown in SiHa cells resulted in a 2.4-fold increase in β-catenin reporter activity (Fig. 4j). Collectively, these results indicated that HOXB4 downregulated Wnt/β-catenin signaling pathway activity.

In addition, the rescue experiment, in which we restored the expression of β-catenin in the HeLa and C-33A cell lines overexpressing HOXB4, reversed the growth inhibitory effect of HOXB4 in vitro and upregulated the expression of the downstream gene c-Myc, without changing HOXB4 (Supplementary Fig. 2f–i). Similarly, transiently transfected 293T cells with β-catenin did not affect either the mRNA level or the protein levels of HOXB4, compared with cells transfection with empty vector control (Supplementary Fig. 2j, k). These data confirmed that HOXB4 inhibited the activity of the Wnt/β-catenin signaling pathway by downregulating β-catenin expression; however, the Wnt/β-catenin signaling pathway had no effect on HOXB4 in cervical cancer cells.

### HOXB4 bound directly to the promoter of β-catenin

**Fig. 5:**
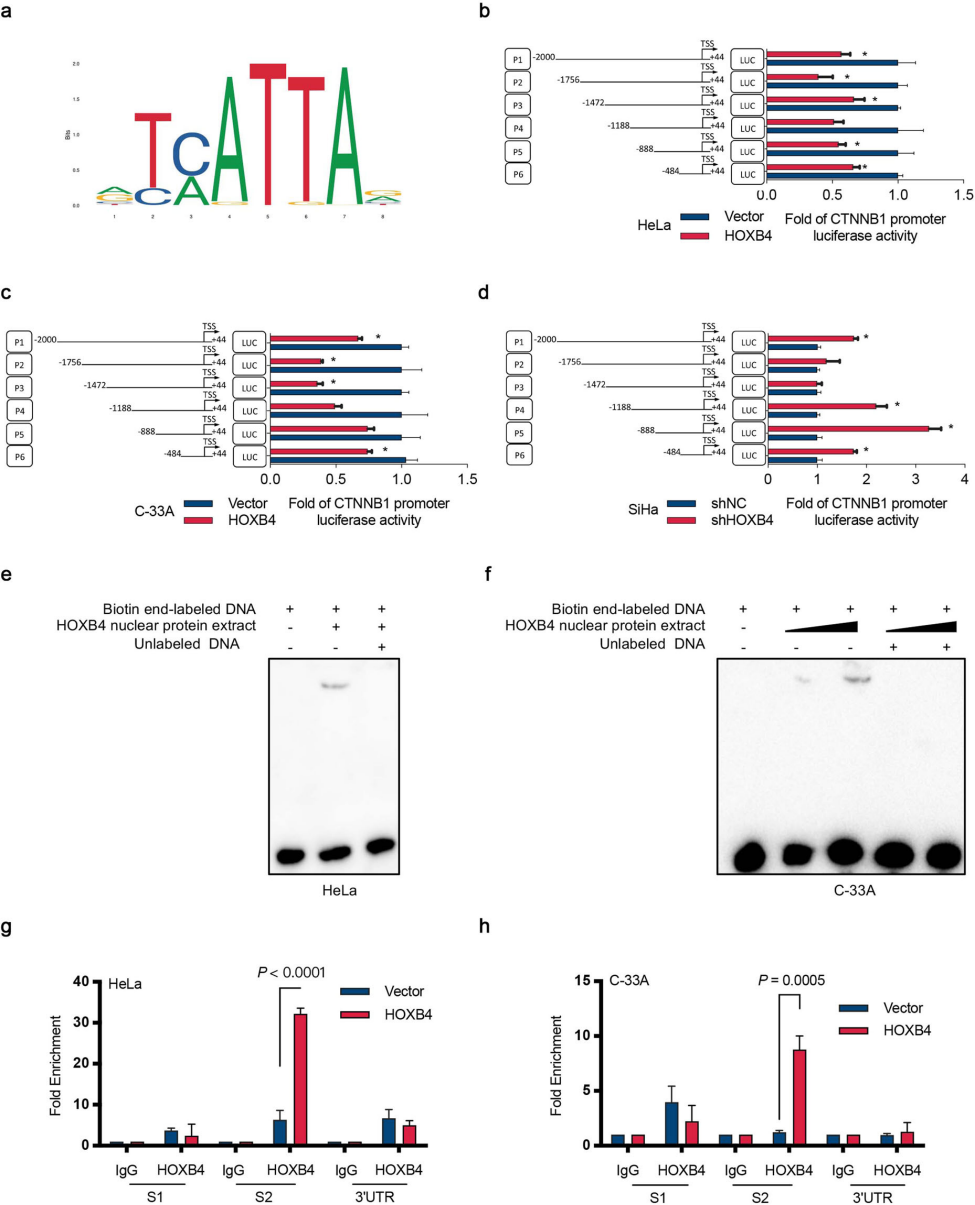
HOXB4 bound to the promoter of β-catenin directly. **a** Transcription factor binding sites of HOXB4. **b**–**d** Luciferase assays assessing the promoter of β-catenin. β-catenin promoter fragments were constructed, and luciferase activity relative to the control was measured in HOXB4-modified HeLa (**b**), C-33A (**c**), and SiHa (**d**) cells. **e**, **f** EMSA assay for the promoter of β-catenin. EMSA was used to detect the direct association of nuclear HOXB4 protein with its binding sites on the promoter regions of β-catenin in HOXB4-overexpressing HeLa (**e**) and C-33A (**f**) cells. **g**, **h** ChIP assays for the promoter of β-catenin. ChIP-qPCR assay in HOXB4-overexpressing HeLa (**g**) and C-33A (**h**) cells were performed with HOXB4 antibody and IgG antibody (negative control).

Analysis of the binding site of HOXB4 using the JASPAR database (http://jaspar.genereg.net/) revealed that the HOXB4 DNA-binding motif contained 5′-TC/AATTA-3′ (Fig. 5a). In addition, the transcription factor binding site search and visualization integrated web tool, the LASAGNA-Search 2.0 (https://biogrid-lasagna.engr.uconn.edu/) showed that HOXB4 bound to the CTNNB1 gene. To test whether HOXB4 transcriptionally regulated β-catenin expression, we searched the β-catenin promoter region of approximately 2 kb (−2000 to +44 bp, relative to the transcription start site [TSS]) in the UCSC genome browser (http://genome.ucsc.edu/), and constructed six promoter reporters (P1-P6) containing different fragments (Fig. 5b). In HeLa and C-33A cells overexpressing HOXB4, the luciferase activity of P6 (−484 to +44 bp) was decreased, whereas in SiHa cells knocking down HOXB4 increased P6 luciferase activity (Fig. 5b–d). In agreement with these findings, overexpression of HOXB4 in 293T cells downregulated the P6 luciferase reporter activity (Supplementary Fig. 3a). Based on the above data, we searched the β-catenin promoter region P6, found the putative HOXB4 binding site, and constructed a mutation of the P6 promoter-reporter (P6M) (Supplementary Fig. 3b). Notably, the mutation of the binding site abolished the HOXB4-mediated reduction in promoter activity (Supplementary Fig. 3c, d). These data suggested that HOXB4 transcription suppressed the expression of β-catenin.

Electrophoretic mobility shift assay (EMSA) was used to determine whether the HOXB4 nuclear protein interacted directly with the putative HOXB4 binding site in HOXB4-overexpressed HeLa and C-33A cells, which was confirmed by the “shift” in migration of the labeled DNA band (Fig. 5e, f). Consistent with our findings, there was a direct interaction between HOXB4 protein and the HOXB4 binding site, 5′-ATAA-3′, in HOXB4-overexpressing 293T cells (Supplementary Fig. 3e). Also, Chromatin immunoprecipitation (ChIP)-qPCR with HOXB4 antibody and IgG (control) indicated enrichment of the β-catenin promoter region S2 (−308 to +44) with the HOXB4 antibody, confirming that HOXB4 inhibited β-catenin transcription by binding to its promoter directly (Fig. 5g, h and Supplementary Fig. 3f). Similar results were observed in 293T cells (Supplementary Fig. 3g). Thus, these data showed that HOXB4 inhibited β-catenin transcription by binding to its promoter at the HOXB4 binding site, 5′-ATAA-3′, directly.

### HOXB4 was negatively correlated with Wnt/β-catenin signaling activity in cervical cancer tissues

**Fig. 6:**
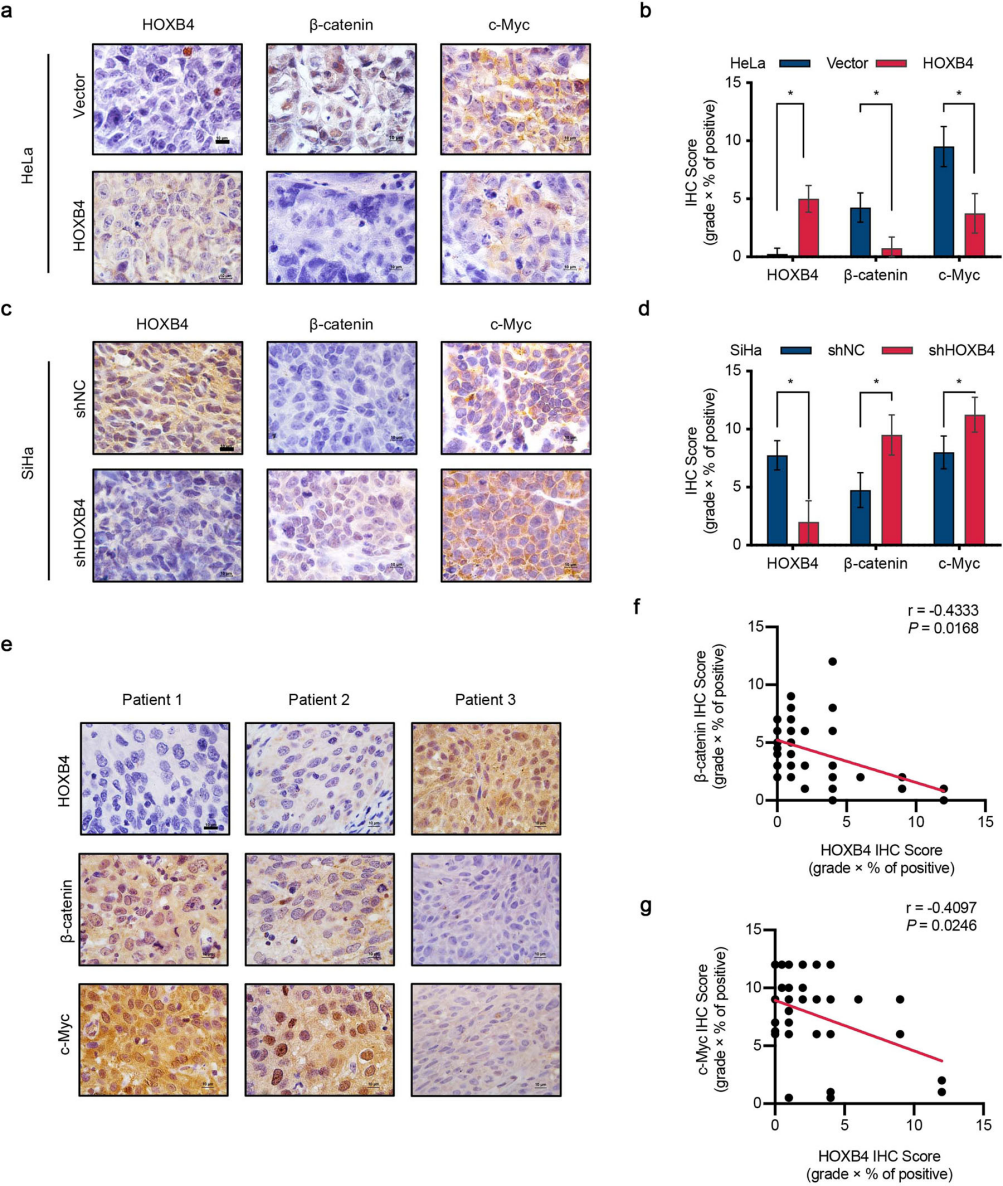
HOXB4 was negatively correlated with β-catenin. **a**–**d** Expression of HOXB4, β-catenin, and c-Myc in vivo. The expression of HOXB4, β-catenin, and c-Myc was measured in the xenograft tumors formed by HOXB4-modified HeLa (**a**, **b**) and SiHa (**c**, **d**) cells using IHC staining. Representative images were shown. Scale bar: 10 μm. **e**–**g** Expression of HOXB4/β-catenin/c-Myc in human cervical cancer. IHC analysis of HOXB4, β-catenin, and c-Myc (**e**) and quantification of IHC scores (**f**, **g**) of human cervical cancer samples. Representative images (**e**) were shown. Scale bar: 10 μm. Correlation between HOXB4 and β-catenin (**f**) or c-Myc (**g)** was calculated. *n* = 30, Pearson chi-square test.

To further confirm the growth inhibitory effect of HOXB4 was related to the activity of the Wnt/β-catenin signaling pathway in vivo, we performed immunohistochemical staining of xenograft tumors with the indicated antibodies. Compared with the control group, the overexpression of HOXB4 significantly reduced the expression of β-catenin and c-Myc (Fig. 6a, b). Conversely, HOXB4 knockdown upregulated the expression of β-catenin and c-Myc, relative to the control group (Fig. 6c, d).

To investigate the relevance of our findings to human cervical cancer, we searched the cervical cancer database from The Cancer Genome Atlas (TCGA) and performed GSEA analysis. We found that LOW group (low expression of HOXB4) was enriched the Wnt/catenin signaling gene set (Supplementary Fig. 4a). Moreover, TCGA data revealed that the expression of HOXB4, β-catenin, and c-Myc showed a significant negative correlation in cervical cancer (Supplementary Fig. 4b, c). The immunohistochemical staining of cervical cancer patient samples further verified the correlation between HOXB4, β-catenin, and c-Myc (Fig. 6e). The IHC score also showed that HOXB4 expression was negatively correlated with β-catenin and c-Myc expression (Fig. 6f, g).

**Fig. 7:**
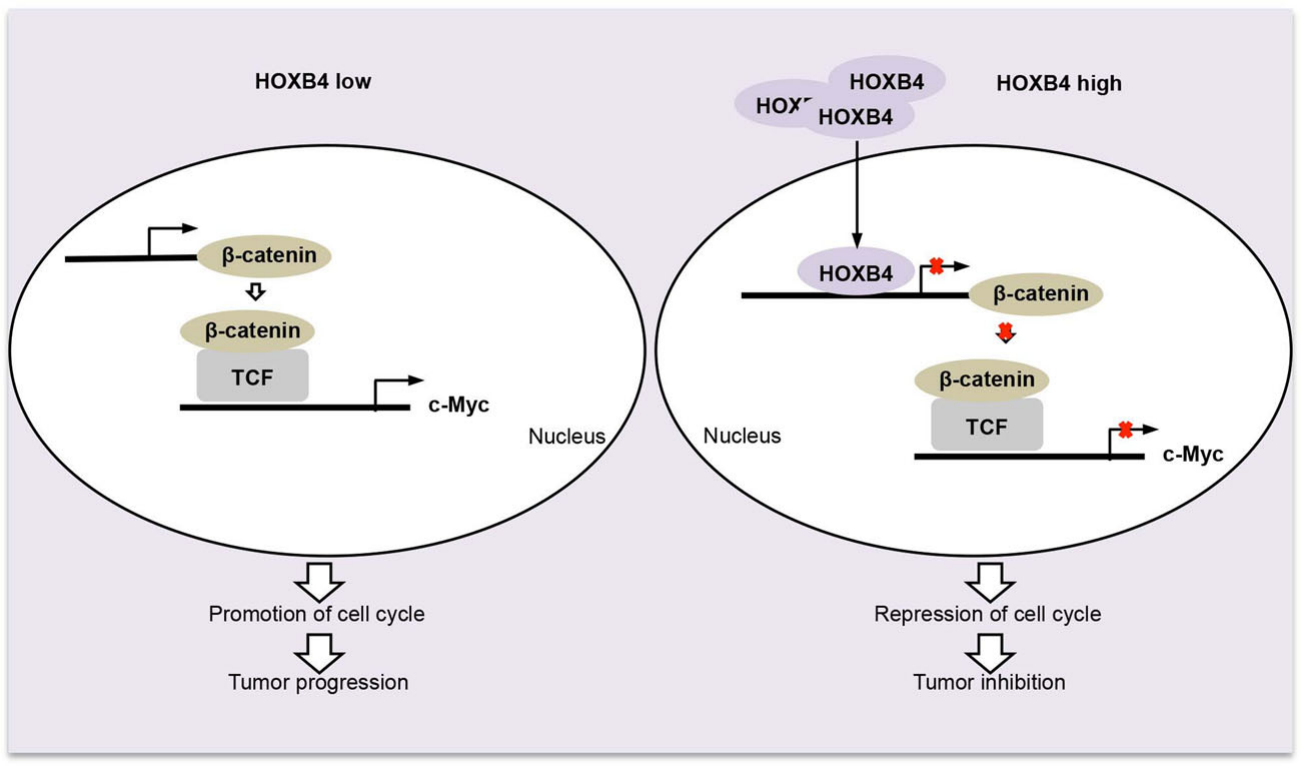
A model of the HOXB4/β-catenin/c-Myc in cervical cancer. In cervical cancer, loss of HOXB4 upregulated β-catenin and its downstream gene c-Myc, which contributed to the enhancement of the cell cycle and promoted tumor progression. In HOXB4-overexpressing cervical cancer cells, HOXB4 was accessed to the nucleus and transcriptionally repressed β-catenin expression, leading to impaired nuclear accumulation of β-catenin, which subsequently contributed to the inhibition of tumor progression.

Collectively, in cervical cancer, the absence of HOXB4 upregulated β-catenin and its downstream gene c-Myc, which contributed to the enhancement of the cell cycle and promoted tumor progression. In HOXB4-overexpressed cervical cancer cells, HOXB4 was accessed to the nucleus and transcriptionally repressed β-catenin expression, leading to impaired nuclear accumulation of β-catenin, which subsequently contributed to the inhibition of tumorigenesis (Fig. 7).

## Discussion

The HOX gene family encodes a class of transcription factors, which have a crucial role in embryonic development. Studies have also shown that their aberrant expression is related to cancers^18,42–44^. HOXB4, a member of the Antp homeobox protein family, regulates embryonic development and participates in tumor progression as an oncogene or tumor suppressor gene. In ovarian cancer, the expression of HOXB4 was closely related to poor prognosis and transcriptionally activated DHDDS to stimulate the proliferation and invasion of tumor cells^23^. Other studies also revealed that HOXB4 was upregulated in ovarian cancer cells and drug-resistant cells^35^. Down-regulation of HOXB4 enhanced the cytotoxic effects of Taxol and DDP on ovarian cancer cells^35^. Therefore, in ovarian cancer, HOXB4 may act as an oncogene. However, the primary role of HOXB4 in breast cancer seems to be tumor suppression. HOXB4 inhibited cell migration and EMT by regulating the STARD13-related signaling pathway^36^. In addition, HOXB4 was downregulated in most breast cancer tissues^36,45^, and its overexpression enhanced sensitivity to doxorubicin^36^. Hence, HOXB4 may serve as a tumor suppressor gene or an oncogene, depending on the specific cancers.

In the present study, we sought to investigate the role and molecular mechanism of HOXB4 in cervical cancer. First of all, our research demonstrated that compared with the normal cervix, HOXB4 was significantly downregulated in cervical cancer, which was inconsistent with previous studies^24–26^. Barba-de detected the expression of HOXB4 in paraffin-embedded tissue microarray in cervical tissues by immunostaining^24^. HOXB4 immunoreactivity was observed in almost all cervical cancer tissues (69/70), but not in normal tissues (0/3)^24^. However, their study only detected HOXB4 expression in three normal cervical epithelium tissues, and the sample size was too small to draw any conclusions. HOXB4 staining was seen in the cytoplasm and/or nucleus of positive cells in normal cervical tissues (Fig. 1c). In normal tissues, the negative staining rate of HOXB4 was 34.1% (14/41) and the positive staining rate was 65.9% (27/41) (Fig. 1d and Supplementary Table 1). In addition, they did not consider the expression of HOXB4 in adjacent tissues, which were positive in all samples (19/19)^24^. Moreover, it is inappropriate to evaluate immunohistochemistry results only by negative and positive staining. In our study, according to the average IHC score (combined staining intensity and positive cell ratio), HOXB4 staining was divided into two categories, negative and positive. The HOXB4 immunoreactivity score revealed that HOXB4 was expressed at different levels and significantly higher than that in cervical cancer (Fig. 1e). Lopez found that HOXB4 was only expressed in 3/11 normal cervical tissues but in the most cervical carcinoma samples (16/17) using RT-PCR^25^. A similar result showed the absence of HOXB4 gene expression in 14 normal cervical tissues^26^. These data were inconsistent with ours. The HOXB4 mRNA expression in 10 normal cervical specimens was 30.6 (9.5–52.7) detected by qRT-PCR, which was higher than the level of 14 specimens of squamous cervical cancer [7.7 (3.3–12.2)] (95% CI, *P* = 0.0155) (Fig. 1g). Furthermore, western blot confirmed HOXB4 was upregulated in normal cervix compared with cervical cancer (Fig. 1f). Secondly, we conducted a series of in vitro and in vivo experiments using cervical cancer cell lines with gain or loss of function of HOXB4, and identified that HOXB4 inhibited cell proliferation and arrested the cell cycle at the transition from G0/G1 phase to S phase. Thus, our findings clearly proved that HOXB4 had as a tumor growth-inhibitor in cervical cancer.

Mechanism studies showed that HOXB4 negatively regulated Wnt/β-catenin signaling pathway. Mutations in the Wnt pathway and its components are responsible for a variety of growth-related pathologies and cancers. Transcription factors of the HOX family have an important role in numerous cellular processes, including cell growth by regulating various signaling pathways, such as the Wnt/β-catenin signaling pathway^46^. Studies on human non-small cell lung cancer (NSCLC) have shown that knocking down HOXB5 significantly inhibited cell proliferation by inhibiting β-catenin and the downstream targets c-Myc and cyclin D1^47^. In the cutaneous squamous cell carcinoma (CSCC) cells, HOXB7 bound to β-catenin, and HOXB7 knockdown reduced cell viability and tumor growth by inhibiting the Wnt/β-catenin signaling pathway^48^. HOXA9 transcriptional activation of WNT6 ultimately activated the canonical WNT/β-catenin pathway in glioblastoma (GBM)^49^. HOX proteins not only promote the development of cancer but also inhibit tumorigenesis by downregulating the Wnt/β-catenin signaling. In lung cancer, HOXA4 was downregulated and inhibited the cell growth by promoting the transcription of GSK3β, thereby inhibiting Wnt signaling^50^. In prostate cancers, HOXB13 downregulated TCF4 and its responsive genes c-Myc and cyclin D1, subsequently inhibiting cell growth^51^. Similarly, in colorectal cancer, HOXB13 downregulated the protein expression of TCF4, thereby hindering the cell growth^52^. Our results showed that HOXB4 directly bound to the promoter of β-catenin at 5′-ATAA-3′, transcriptionally inhibited its expression and subsequently suppressed the activity of the Wnt/β-catenin signaling pathway, which was consistent with previous studies^42^ that the homeodomain (evolutionally conserved 60-amino-acid long DNA-binding domain) only recognized and bound to four base-pair sequences (TAAT/ATTA/ TTAT/ATAA).

GESA analysis of the data from TCGA-CESC confirmed that HOXB4 inhibited the activity of the Wnt/β-catenin signaling pathway and correlation analysis showed that in cervical cancer, the expression of HOXB4, β-catenin, and c-myc genes were significantly negatively correlated (Supplementary Fig. 4). However, the clinical correlation between HOXB4 and β-catenin gene expression in TCGA-CESC was low (*r* = −0.1243, *P* < 0.05). There may be two explanations. First, the patient’s gene expression was affected by many factors, such as pathological type, pathological stage, age, drug therapy, basic gene expression level, etc. These factors will affect the expression of mRNA. Second, the pathological tissue of the patient we obtained may include multiple cell types, such as cervical cancer cells, stromal cells, immune cells, etc., which may affect the correlation between the two genes in cancer tissues.

In summary, we found that HOXB4 was markedly downregulated in cervical cancer. A series of in vitro and in vivo experiments proved that HOXB4 functioned as a growth-inhibition role in cervical cancer. Mechanistically, HOXB4 inhibited cervical tumorigenesis by repressing the transcription of β-catenin, thereby inhibiting Wnt/β-catenin signaling pathway activity.

## Supplementary information

Supplementary Figure 1

Supplementary Figure 2

Supplementary Figure 3

Supplementary Figure 4

Supplementary Table 1

Supplementary Table 2

Supplementary Table 3

Supplementary Table 4

Supplementary Table 5

## Supplementary information

**Supplementary Information** The online version contains supplementary material available at 10.1038/s41419-021-03411-6.
