# Supplementary figures and images for: HOXB4 inhibits the proliferation and tumorigenesis of cervical cancer cells by downregulating the activity of Wnt/β-catenin signaling pathway
Source: Cell Death Dis. 2021 Jan 21;12(1):105. doi: 10.1038/s41419-021-03411-6 (PMC7820415; doi:10.1038/s41419-021-03411-6)

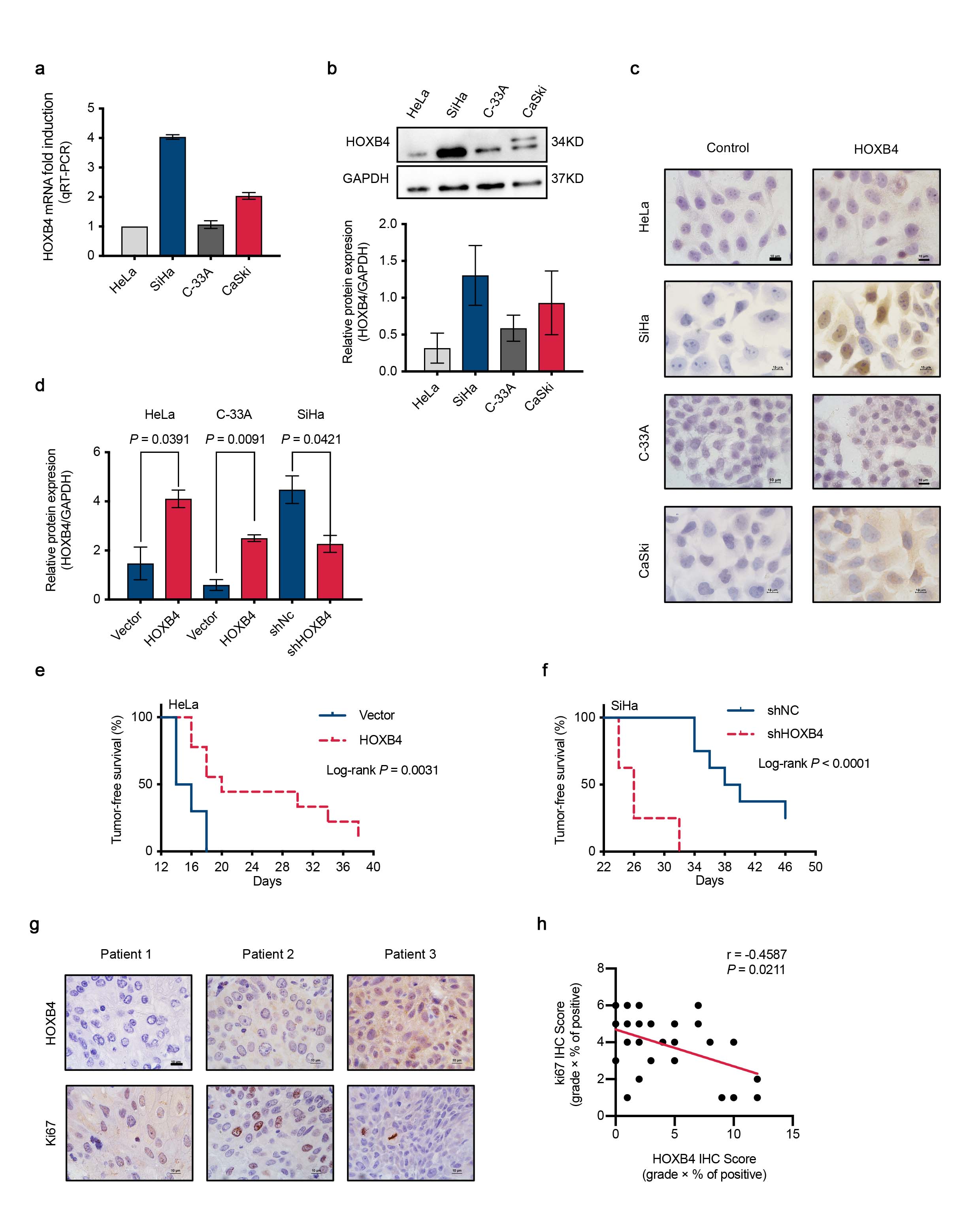

Supplement: Supplementary file 1 — Supplementary Figure 1 [file 41419_2021_3411_MOESM1_ESM.jpg]

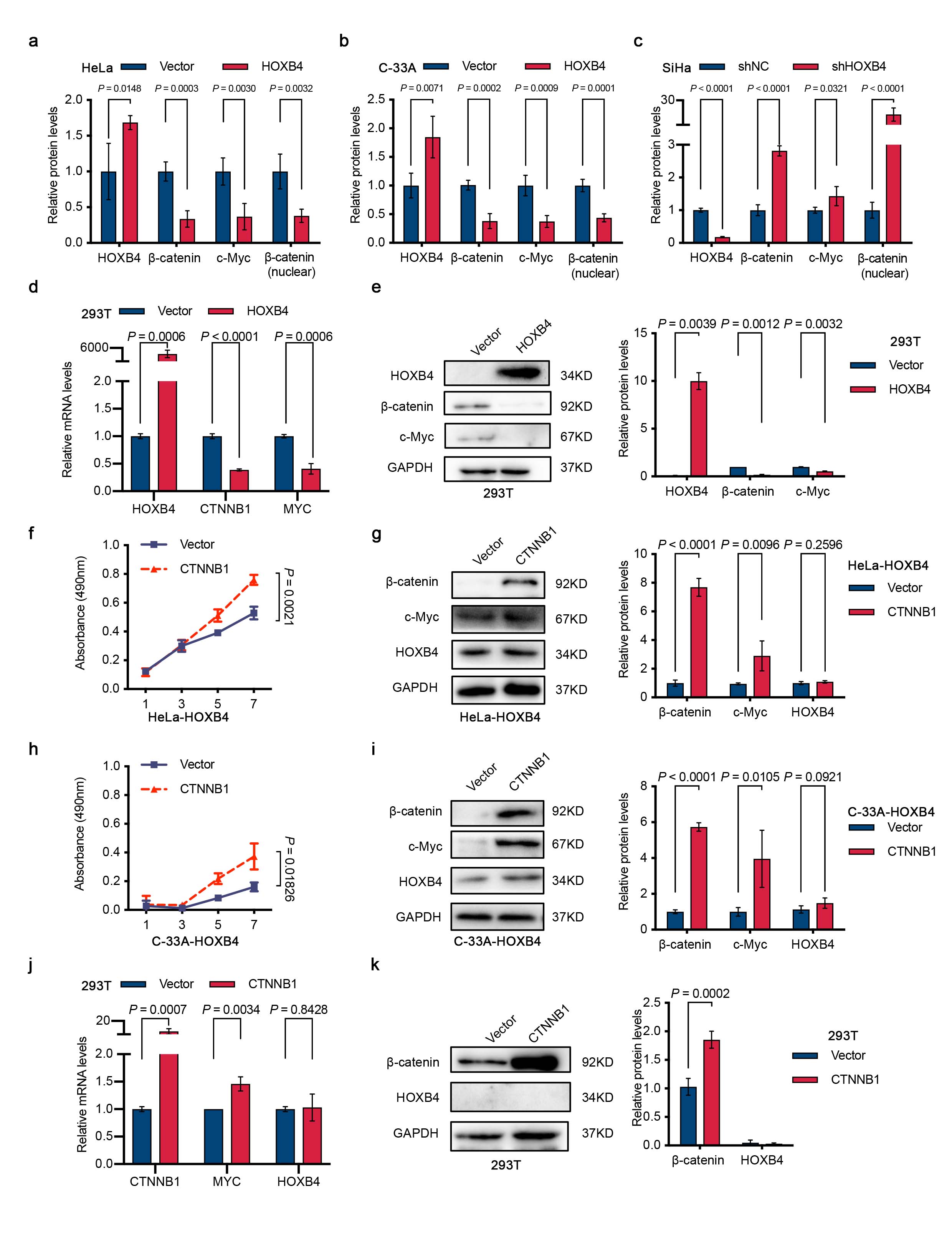

Supplement: Supplementary file 2 — Supplementary Figure 2 [file 41419_2021_3411_MOESM2_ESM.jpg]

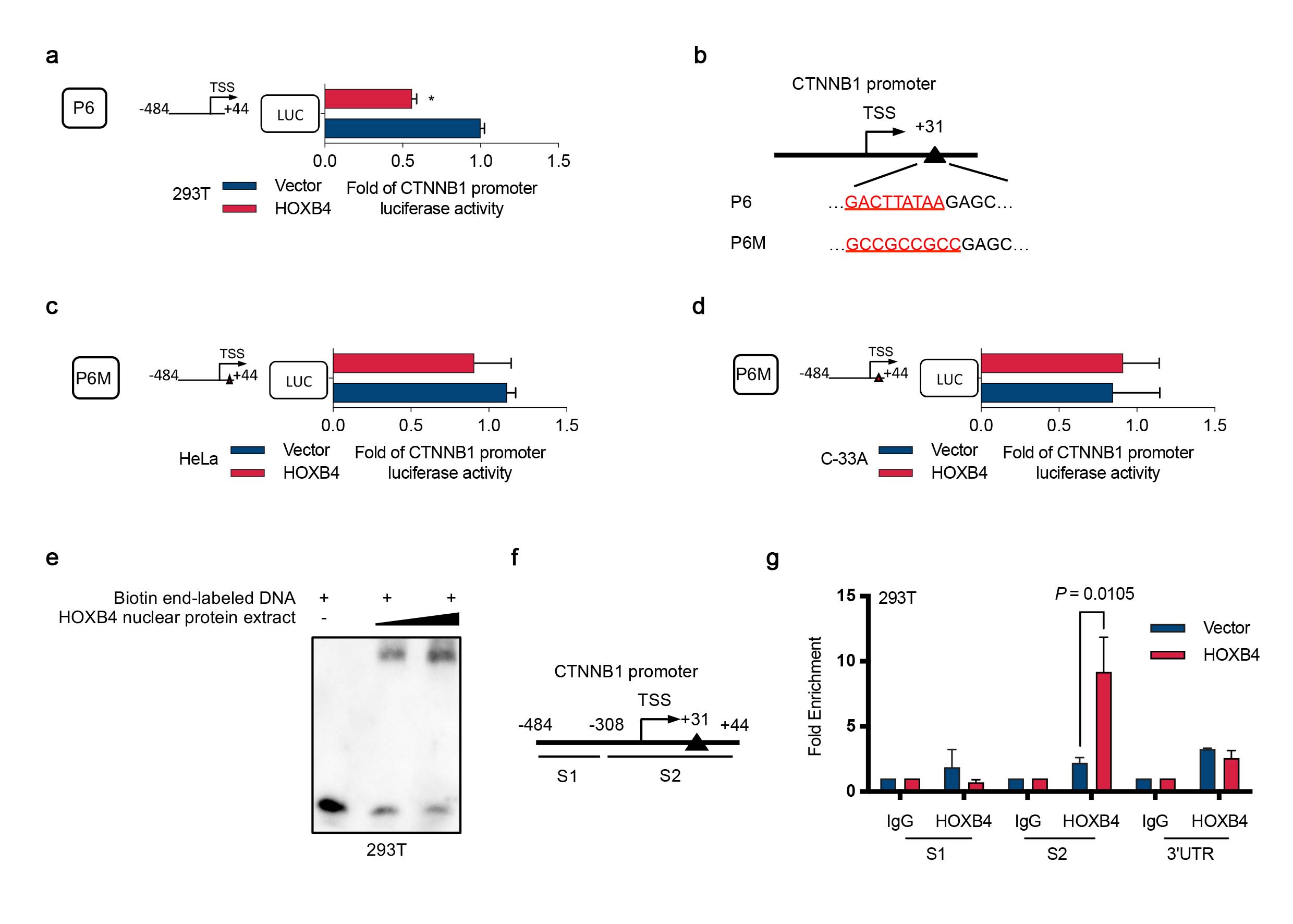

Supplement: Supplementary file 3 — Supplementary Figure 3 [file 41419_2021_3411_MOESM3_ESM.jpg]

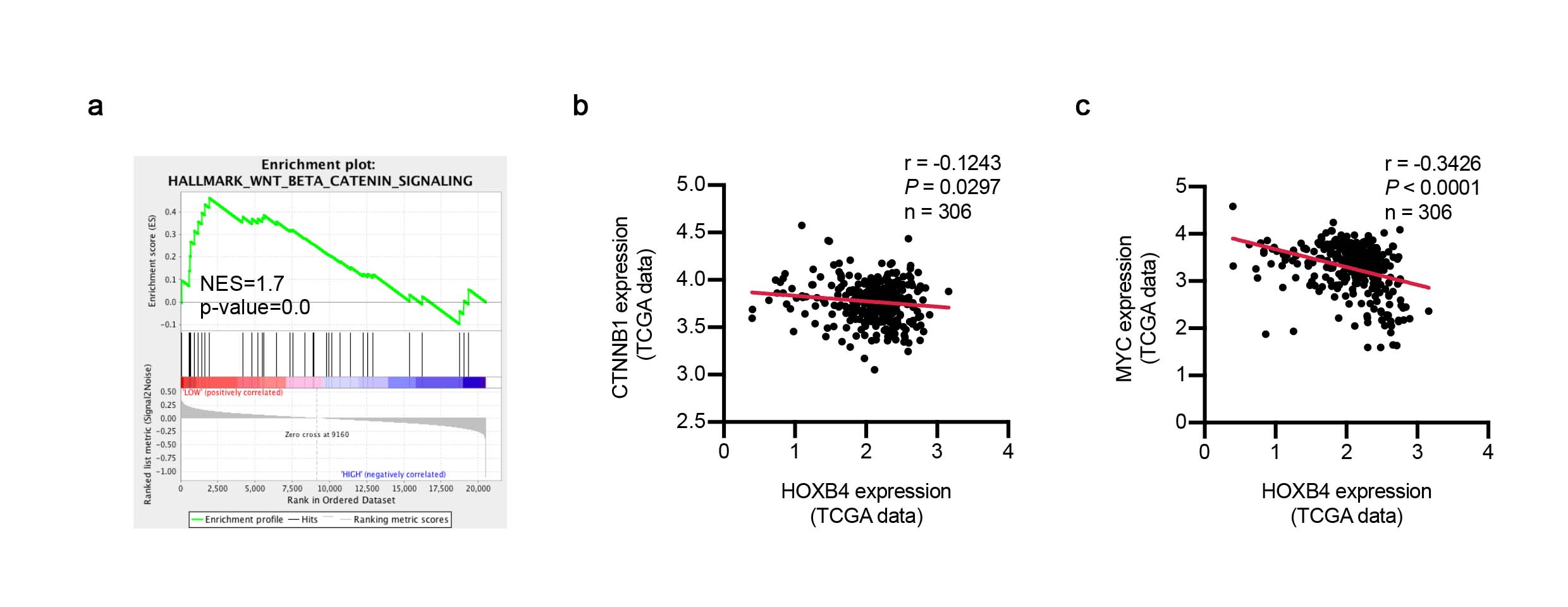

Supplement: Supplementary file 4 — Supplementary Figure 4 [file 41419_2021_3411_MOESM4_ESM.jpg]
